# Phospholipase D regulates the size of skeletal muscle cells through the activation of mTOR signaling

**DOI:** 10.1186/1478-811X-11-55

**Published:** 2013-08-02

**Authors:** Rami Jaafar, Joffrey De Larichaudy, Stéphanie Chanon, Vanessa Euthine, Christine Durand, Fabio Naro, Philippe Bertolino, Hubert Vidal, Etienne Lefai, Georges Némoz

**Affiliations:** 1 Lyon 1 University, INSERM U1060, CarMeN Laboratory, Institut National de la Recherche Agronomique USC1235, F-69600 Oullins, France; 2 Istituto Interuniversitario di Miologia and Dipartimento di Istologia ed Embriologia Medica, Università di Roma-La Sapienza, 00161, Roma, Italy; 3 Centre de Recherche en Cancérologie de Lyon, INSERM U1052, CNRS UMR 5286, 69008 Lyon, France

**Keywords:** Phospholipase D, Phosphatidic acid, Muscle wasting, Muscle hypertrophy, Myotubes, mTOR

## Abstract

mTOR is a major actor of skeletal muscle mass regulation in situations of atrophy or hypertrophy. It is established that Phospholipase D (PLD) activates mTOR signaling, through the binding of its product phosphatidic acid (PA) to mTOR protein. An influence of PLD on muscle cell size could thus be suspected. We explored the consequences of altered expression and activity of PLD isoforms in differentiated L6 myotubes. Inhibition or down-regulation of the PLD1 isoform markedly decreased myotube size and muscle specific protein content. Conversely, PLD1 overexpression induced muscle cell hypertrophy, both *in vitro* in myotubes and *in vivo* in mouse gastrocnemius. In the presence of atrophy-promoting dexamethasone, PLD1 overexpression or addition of exogenous PA protected myotubes against atrophy. Similarly, exogenous PA protected myotubes against TNFα-induced atrophy. Moreover, the modulation of PLD expression or activity in myotubes showed that PLD1 negatively regulates the expression of factors involved in muscle protein degradation, such as the E3-ubiquitin ligases Murf1 and Atrogin-1, and the Foxo3 transcription factor. Inhibition of mTOR by PP242 abolished the positive effects of PLD1 on myotubes, whereas modulating PLD influenced the phosphorylation of both S6K1 and Akt, which are respectively substrates of mTORC1 and mTORC2 complexes. These observations suggest that PLD1 acts through the activation of both mTORC1 and mTORC2 to induce positive trophic effects on muscle cells. This pathway may offer interesting therapeutic potentialities in the treatment of muscle wasting.

## Lay abstract

The phospholipase D (PLD) enzyme transforms phosphatidylcholine, a major lipid constituent of cell membranes, into a messenger endowed with many activities in the cell. PLD is known to influence the activity of mTOR, a signaling pathway that plays an important role in muscle mass regulation. We thus researched whether PLD had an effect on the size of cultured muscle cells. To this end, we used various types of PLD inhibitors, as well as systems allowing to modify PLD expression. We observed that both PLD inhibition and decreased expression induced muscle cell atrophy, associated with an increased expression of factors involved in protein degradation. Conversely, overexpressing PLD induced a hypertrophy and a decreased expression of these factors. We further demonstrated that the changes in muscle cell size induced by PLD were mediated by mTOR. This study establishes that PLD has a positive influence on muscle cells, and suggests that it could be a target in therapeutic interventions aiming at preserving muscle tissue from wasting associated with chronic diseases.

## Background

Phospholipase D (PLD) catalyzes the conversion of the membrane phospholipid phosphatidylcholine into the messenger phosphatidic acid (PA). Two isoforms of PLD have been identified, PLD1 and PLD2, each of which exhibiting specific regulatory properties and subcellular localization [1,2]. This enzyme has been extensively studied for its implication in vesicular trafficking, cytoskeletton dynamics, cell migration, survival, differentiation and proliferation [3]. Since the pioneer work of Chen’s group [4,5], its involvement in mTOR (mammalian Target Of Rapamycin) signaling has attracted an increasing interest. mTOR senses and integrates a variety of environmental cues to regulate major cellular processes [6]. The ability of PLD and its product PA to activate mTOR signaling through both mTORC1 and mTORC2 complexes has been widely described [7,8]. PA was shown to bind to the FRB domain of mTOR protein, in competition with the complex that the selective mTOR inhibitor rapamycin forms with the immunophilin FKBP12 [4,9-11]. PA was also shown to stimulate mTORC1 kinase activity by displacing the FKBP38 inhibitor and by exerting direct effects on mTOR [11]. Furthermore, it has been reported that PA binding is required for the assembly of both mTORC1 and mTORC2 complexes, with a higher apparent PA affinity for the latter [10]. The role of PLD in the activation of mTOR pathway is also supported by a number of studies. The ability of the small G protein Rheb, a key regulator upstream of mTORC1, to bind and activate PLD1 in a GTP-dependent manner supports the contribution of PLD1 to mTORC1 signaling as an effector of Rheb [12]. Furthermore, whereas amino-acids stimulate PLD activity and induce PLD1 translocation to the vicinity of mTOR [13], PLD1 depletion or PLD1/2 inhibition impair amino-acid dependent mTORC1 activity [13,14].

The contribution of PLD and PA to mTOR signaling is expected to be particularly relevant in skeletal muscle, in which mTOR is thought to play a crucial role in tissue adaptation to changes in physiological and pathological conditions. Thus, muscle hypertrophic stimuli such as mechanical loading, feeding, IGF-I, activate mTORC1 signaling, whereas it is inhibited by atrophic stimuli such as unloading, starvation and glucocorticoids (reviewed in [15]). Rapamycin inhibition of hypertrophic responses further supports the involvement of mTORC1 in muscle hypertrophy [16]. Accordingly, mechanical loading-induced hypertrophy is preserved under rapamycin treatment in transgenic mice expressing a rapamycin-resistant form of mTOR specifically in muscle [17]. The anabolic actions of mTORC1 are related to its ability to activate protein synthesis by enhancing translation initiation and elongation, to upregulate ribosome and mitochondrial biogenesis, and to negatively regulate autophagy [6]. Accordingly, transgenic mice selectively lacking mTOR [18] or mTORC1 [19] in skeletal muscle develop a severe dystrophy accompanied by a myofibre atrophy.

We and others previously reported the involvement of PLD in myogenic differentiation, suggesting that this enzyme is important for muscle development [20-23]. Moreover, a role for PLD in mechanically-induced muscle hypertrophy was hypothesized, as stretches imposed on mouse isolated EDL muscles induced a sustained PLD-dependent accumulation of PA, resulting in mTORC1 stimulation [24]. Similarly, EDL muscles submitted to eccentric contractions showed stably increased PA levels. Interestingly, PA accumulation preceded a PI3 kinase/Akt independent activation of mTORC1 that could be prevented by 1-butanol, an inhibitor of PA production by PLD [25]. However, despite these evidences a direct demonstration of PLD implication in the regulation of muscle cell size remains to be provided. Thus, we set out to investigate the effects that modulation of PLD activity or expression exerts on the size and functional parameters of differentiated L6 myotubes, submitted or not to atrophy-inducing treatments. We found that PLD participated in trophic responses of muscle cells in culture, and observed an *in vivo* hypertrophic effect of increased PLD expression. We then investigated the consequences of alterations in PLD activity on mTOR signaling pathway, and found that both mTORC1 and mTORC2 are modulated by PLD and may participate in the trophic responses we observed in L6 myotubes. Thus, our results support the view that targeting PLD could represent a novel way to influence muscle mass.

## Results

### Changes in PLD activity have trophic effects on muscle cells

**Figure 1:**
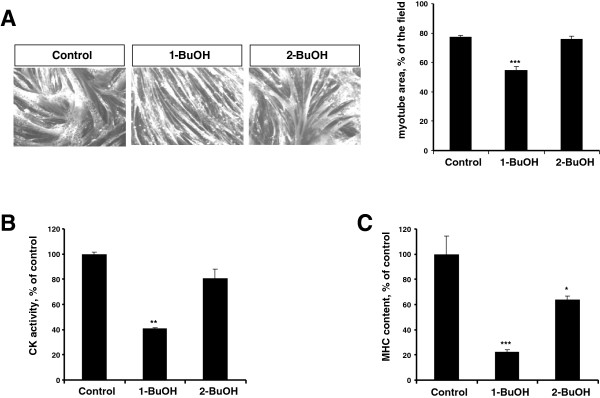
**Atrophic effects of 1-butanol on L6 myotubes.** Differentiated L6 myotubes were cultured for 2 days in the presence of 0.5% 1-butanol, or 0.5% 2-butanol, or left untreated (control). **(A)** Myotubes were immuno-stained with anti-MHC antibody, and myotube area was measured as reported in [30]. Data are means ± SE of n = 8. **(B)** Myotubes were homogenized and creatine kinase activity was measured. Data are means ± SE of n = 3. **(C)** MHC content of myotubes was assessed by ELISA. Data are means ± SE of n = 3. ***: different from control, p < 0.001, **: p < 0.01, *: p < 0.05.

**Figure 2:**
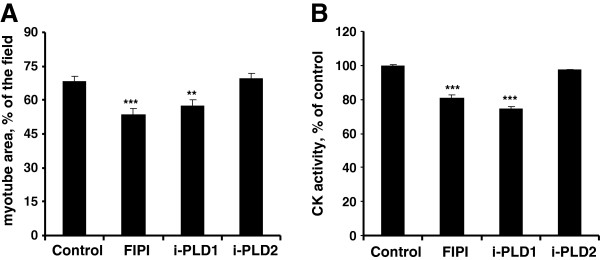
**Atrophic effects of PLD inhibitors on L6 myotubes.** Differentiated L6 myotubes were cultured for 2 days without inhibitor (control), or in the presence of 0.5 μM FIPI, or 100 nM PLD1-inhibitor, or 100 nM PLD2-inhibitor. **(A)** Myotube area was measured as above. Data are means ± SE of n = 10. **(B)** Myotubes were homogenized and creatine kinase activity was measured. Data are means ± SE of n = 4. ***: different from control, p < 0.0001; **: p < 0.01.

**Figure 3:**
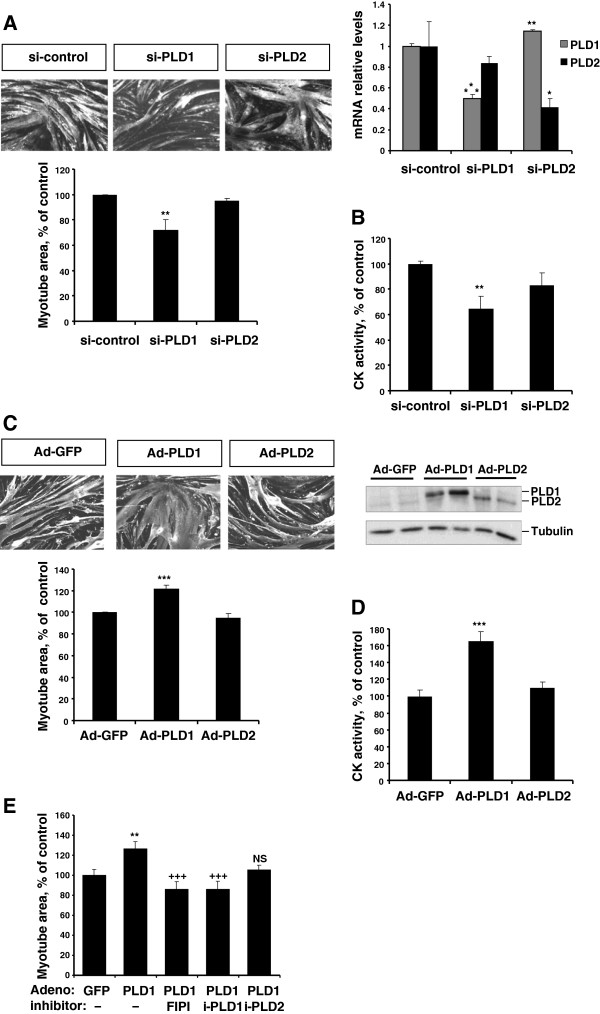
**The modulation of PLD expression has trophic effects in L6 myotubes. (A)** Effects of siRNA-mediated PLD depletion on the area of differentiated myotubes. An irrelevant siRNA was used for control. Results shown are the means ± SE of 3 experiments, with 10 repeats. **: different from control siRNA, p < 0.01. The down-regulation of PLD isoforms was verified by RT-qPCR (right panel). Means ± SE of n = 3 or 4 are shown. ***: different from control siRNA, p < 0.0001; **: p < 0.01; *: p = 0.05. **(B)** Effects of siRNA-mediated PLD depletion on CK activity of differentiated myotubes. Means ± SE of n = 4 are shown. **: different from control siRNA-treated cells, p < 0.01. **(C)** Effects of adenovirus-mediated PLD overexpression on myotube area. An adenovirus encoding GFP was used for control. Shown are the means ± SE of 8 experiments with 10 replicates. ***: different from GFP-adenovirus infected cells, p < 0.0001. The overexpression of PLD isoforms was verified by immunoblotting of HA-tagged PLDs (right panel). **(D)** Effects of Adenovirus-mediated PLD overexpression on myotube CK activity. Means ± SE of n = 5. ***: different from GFP-adenovirus infected cells, p < 0.001. **(E)** Effect of PLD inhibition on the hypertrophic response of myotubes overexpressing PLD1. Myotubes were infected with GFP- or PLD1-adenovirus and simultaneously treated with PLD inhibitors as detailed in Figure 2 legend. Results are shown as means ± SE of 10 to 20 replicates. **: different from GFP-adenovirus infected cells, p = 0.002; ^**+++**^: different from PLD1-adenovirus infected cells, p < 0.001; NS: not significantly different from PLD1-adenovirus infected cells.

We first addressed the contribution of PLD to the maintenance of muscle cell functionality by studying the consequences of PLD inhibition in fully differentiated L6 myotubes. Preventing PA formation by PLD can be achieved by the addition of a primary alcohol that reroutes PLD activity to the production of phosphatidylalcohol. Myotubes were treated for 48 hrs with either 0.5% 1-butanol, or 0.5% 2-butanol that is not recognized by PLD and serves as a negative control. Immunofluorescent labelling of myosin heavy chain (MHC) was subsequently used to measure myotube area. 1-butanol induced a marked decrease of myotube area, whereas 2-butanol had no significant effects (Figure 1A). Creatine kinase (CK) activity of treated myotubes was also determined to evaluate muscle cell functionality. 1-butanol had a stronger negative effect on myotube CK activity than 2-butanol (Figure 1B). In addition, MHC content of myotubes was found more markedly lowered by 1-butanol than by 2-butanol (Figure 1C). These results suggest that inhibiting PLD activity induces an atrophy of myotubes, that is reflected by a decreased cell size and a loss of muscle proteins. Because concerns have been raised about the effect of primary alcohols as an index of PLD involvement in cell responses [26,27], we assessed the effects of small molecule inhibitors of PLD. Treatment of myotubes by FIPI, an inhibitor of both PLD isoforms [28], resulted in a marked atrophy, thereby confirming the involvement of PLD inhibition in the above observations (Figure 2A,B). We then used PLD isoform-specific inhibitors [29], and observed that PLD1 inhibition affected myotube chatacteristics, whereas PLD2 inhibition had no significant effect (Figure 2A,B). Finally, the respective role of PLD isoforms was further assessed by using PLD1- or PLD2-siRNA. This approach confirmed that PLD1 depletion was more efficient than PLD2 depletion to decrease myotube area and CK activity (Figure 3A,B). Conversely, we found adenovirus-mediated overexpression of PLD1 to significantly increase myotube area and CK activity as compared with control cells, whereas PLD2 overexpression had no significant effect (Figure 3C,D). These observations confirmed that PLD1 positively regulates muscle cells. To verify that enzymatic activity is required for PLD1 trophic effects, we treated PLD1-overexpressing myotubes with PLD inhibitors. As expected, the dual PLD inhibitor FIPI and the PLD1-specific inhibitor both suppressed the hypertrophy induced by PLD1 overexpression, whereas the PLD2-specific inhibitor had no sigificant effect (Figure 3E).

**Figure 4:**
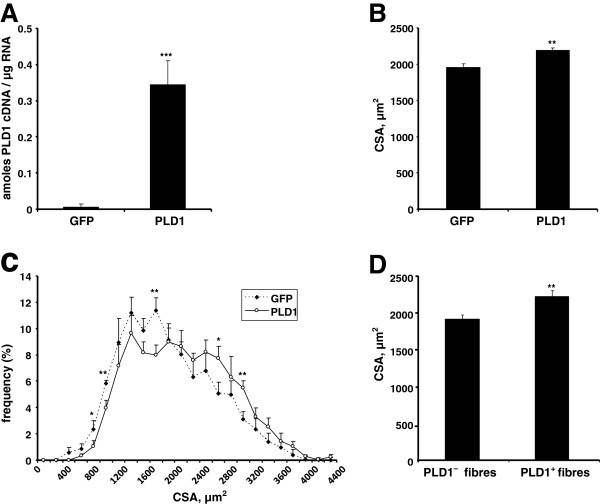
**PLD1 overexpression in mice results in muscle hypertrophy.** Adenovirus encoding PLD1 or GFP were respectively injected in the right and the left gastrocnemius of mice. **(A)** PLD1 overexpression was assessed by RT-qPCR of hPLD1 performed in both GFP- and PLD1-injected gastrocnemius muscles. Means ± SE of n = 5 are shown. ***: p < 0.0001. **(B)** Myofibre CSA measurements from transverse sections of gastrocnemius muscles injected with either GFP-adenovirus or PLD1-adenovirus. The mean CSA ± SE of 5 animals were compared by a paired *t*-test. **: p = 0.01. **(C)** Myofibre CSA distributions in GFP-adenovirus injected and PLD1-adenovirus injected muscles. Means ± SE of n = 5 are shown. **: p < 0.01; *: p < 0.05. **(D)** CSA of HA-PLD1 expressing fibres were compared to the CSA of non-PLD1 expressing fibres in sections of right gastrocnemius. **: p < 0.01 (means ± SE of 247 PLD1^-^ and 149 PLD1^+^ counted fibres).

Next, we assessed the *in vivo* relevance of these observations. We injected a PLD1-encoding adenovirus in the right gastrocnemius of mice, the left gastrocnemius being injected with an adenovirus encoding GFP as a control. Muscles were dissected 10 days following injection, and PLD1 overexpression was verified (Figure 4A). Measurement of myofibre cross sectional area (CSA) demonstrated a significant increase in myofibre size in PLD1-injected muscles as compared with GFP-injected ones, as shown by a shift of the CSA distribution curve towards higher values (Figure 4B,C). Taking advantage of the HA-tag fused to our PLD1-expressing construct, we then compared the respective CSA of myofibres expressing or not the fusion protein, in sections of PLD1-injected muscles. Immunofluorescent labeling of recombinant HA-tagged PLD1 (Additional file 1) followed by CSA measurement confirmed a significant increase (16%) in the size of PLD1-expressing fibres (Figure 4D).

### PLD and PA counteract the atrophic response of myotubes induced by catabolic agents

**Figure 5:**
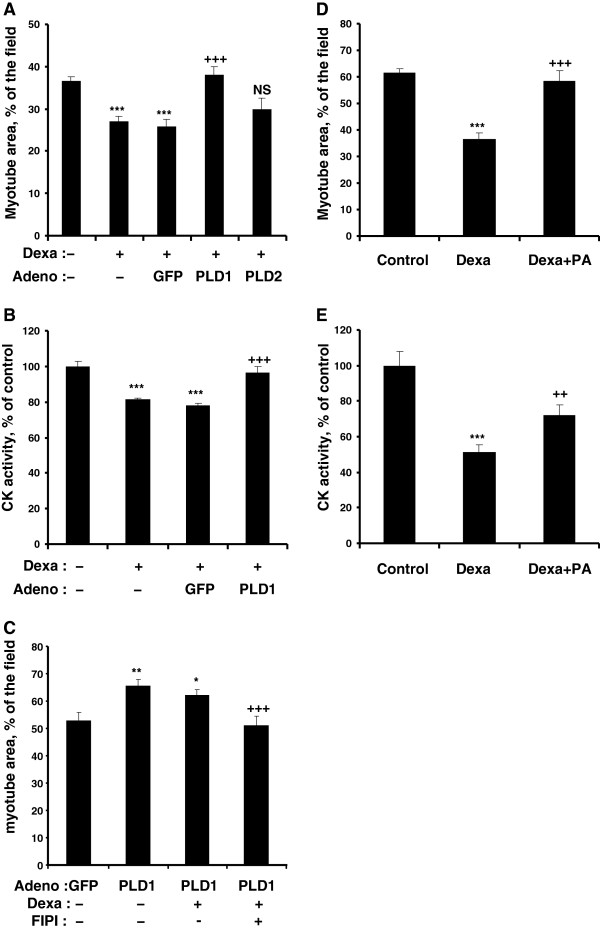
**PLD and PA protect L6 myotubes from dexamethasone-induced atrophy. (A)** Differentiated myotubes were left untreated, or were treated with 20 μM dexamethasone alone or in the presence of GFP-, or PLD1-, or PLD2-adenovirus for 2 days. Myotube area was then assessed. Results are shown as means ± SE of n = 10. ***: different from control, p < 0.001. ^**+++**^: different from dexamethasone and GFP-adenovirus treated cells, p < 0.001; NS: not significantly different from dexamethasone and GFP-adenovirus treated cells. **(B)** Creatine kinase activity was measured in differentiated myotubes treated as above. Means ± SE of n = 4 replicates are shown. ***: different from control, p < 0.001; ^**+++**^: different from GFP-adenovirus infected cells treated with dexamethasone, p < 0.001. **(C)** Myotubes were infected with GFP- or PLD1-adenovirus and simultaneously treated with dexamethasone as above, in the presence or absence of FIPI. Myotube area was measured and results are shown as means ± SE of n = 5 to 10. **: different from GFP-adenovirus infected cells, p < 0.01; *: p < 0.05; ^**+++**^: different from PLD1-adenovirus infected cells, p < 0.001. **(D)** Myotubes were treated for 2 days with 100 μM dexamethasone in the presence or absence of 100 μM PA, and myotube area was assessed. Means ± SE of n = 6 to 8 are shown. ***: different from control, p < 0.0001; ^**+++**^: different from dexamethasone treated cells, p < 0.0001. **(E)** CK activity was measured in myotubes treated as above. Means ± SE of n = 4 are shown. ***: different from control, p < 0.0001; ^**++**^: different from dexamethasone treated cells, p < 0.01. Note that a lower dexamethasone concentration was used in the experiments of panels A, B, C, to prevent an excessive stress of the cells submitted to an adenoviral infection.

**Figure 6:**
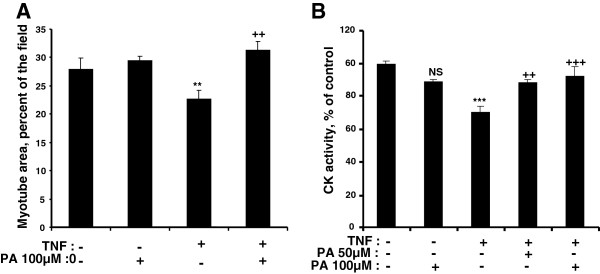
**PA protects L6 myotubes from TNFα-induced atrophy. (A)** Differentiated myotubes were left untreated, or treated with 100 μM PA, or with a combination of 15 ng/ml TNFα and 100 μM PA. Myotube area was then assessed. Results are shown as means ± SE of n = 10 fields. **(B)** Myotubes left untreated, or treated with 100 μM PA, or with a combination of 15 ng/ml TNFα and 50 or 100 μM PA, were used to measure CK activity. Means ± SE of n = 6 replicates are shown. ***: different from control, p < 0.0001; **: p = 0.02; NS: not significant. ^**+++**^: different from TNFα treated cells, p < 0.0001; ^**++**^: p < 0.001.

Muscle cell atrophy can be induced *in vivo* and *in vitro* by synthetic glucocorticoids such as dexamethasone [31,32]. We investigated the effects of PLD isoform overexpression in dexamethasone-treated myotubes. As expected, dexamethasone induced a marked atrophy of myotubes, as evidenced by reduced myotube size and CK activity. Interestingly, this atrophic effect was suppressed in PLD1-overexpressing cells, but not affected by PLD2 overexpression (Figure 5A,B). Moreover, inhibition of PLD activity by FIPI restored the atrophic effect of dexamethasone in PLD1-overexpressing myotubes (Figure 5C). Next, we mimicked PLD activation by adding exogenous PA to dexamethasone-treated cells. We found PA addition able to partially restore both myotube size and CK activity (Figure 5D,E). We then used another agent able to induce atrophy of muscle cells, the pro-inflammatory cytokine TNFα [33,34]. We observed that the addition of exogenous PA suppressed the negative effects of TNFα on both myotube size and CK activity (Figure 6A,B). Taken together, these data demonstrate that both PLD1 overexpression and exogenous PA supply had an anti-atrophic effect, in the presence of two different atrophy-inducing treatments.

### Modulation of PLD activity affects the expression of atrogenes

**Figure 7:**
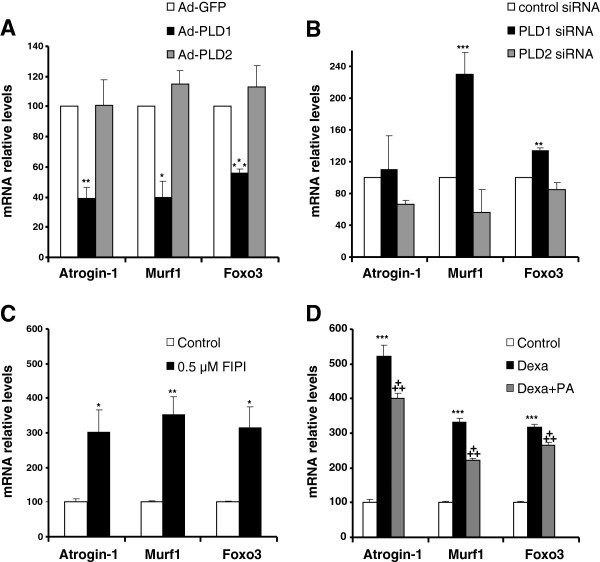
**PLD modulates the expression of atrogenes in myotubes. (A)** Effects of adenovirus-mediated PLD overexpression on atrogene mRNA levels. GFP-adenovirus infected myotubes were used for control. **(B)** Effects of siRNA-mediated PLD depletion on the expression of atrogenes. An irrelevant siRNA was used for control. **(C)** Effects of PLD inhibition by FIPI on the expression of atrogenes. Myotubes were left untreated or were treated with 0.5 μM FIPI for 2 days. **(D)** Effect of the addition of 100 μM exogenous PA on the expression of atrogenes in the presence of 100 μM dexamethasone. Control cells received no treatment. All the mRNA levels were normalized to the levels of TBP mRNA in the samples. For all experiments, results are shown as means ± SE of n = 4 to 6 replicates. ***: different from control, p ≤ 0.001; **: p < 0.01; *: p < 0.05. ^**+++**^: different from dexamethasone treated cells, p < 0.001.

Muscle atrophy is closely related to changes in the expression of a set of genes called atrogenes [35], that include the E3 ubiquitin ligases Murf1 and Atrogin-1 involved in the proteasome-dependent muscle protein catabolism [36]. Cell proteolytic systems are under the positive control of Foxo transcription factors, in particular Foxo3 [37]. To get insight into PLD action on muscle proteolytic machinery, we assessed the expression of Murf1, Atrogin-1 and Foxo3 transcripts in L6 myotubes subjected to PLD modulation. As shown in Figure 7A, we observed a strong inhibition of the basal expression of the three genes specifically in cells overexpressing PLD1, but not in PLD2-overexpressing cells. Furthermore, the siRNA-mediated depletion of PLD1 induced a marked increase in Murf1 and Foxo3 expression, whereas the down-regulation of PLD2 had no significant effect (Figure 7B). From here we deduced that PLD1 hypertrophic effects may be related to its capacity to down-regulate the basal expression of genes involved in proteolysis. To confirm the role of PLD in the negative control of atrogene expression, we then treated myotubes with the PLD inhibitor FIPI. We observed that PLD inhibition markedly increased atrogene mRNA levels (Figure 7C). We next evaluated the effects of a PA treatment on atrogene expression induced by dexamethasone. In agreement with its pro-atrophic properties, we found dexamethasone to induce a robust expression of the atrogenes. However, these effects were significantly lowered by the addition of exogenous PA (Figure 7D). On the whole, these observations show that PLD and PA are able to down-regulate atrogene expression, both in basal conditions and in dexamethasone-induced atrophy.

### PLD1 effects on muscle cells are mediated by mTOR

**Figure 8:**
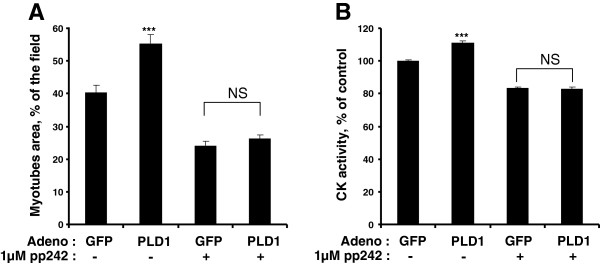
**Inhibition of mTOR suppresses the hypertrophic effects of PLD1. (A)** Myotubes were infected with GFP-adenovirus, or with PLD1-adenovirus, and were subsequently treated or not with 1 μM PP242 for 48 hrs. Myotube area values are means ± SE of n = 10 to 20 fields. **(B)** CK activity was measured in myotubes treated as above. Results are shown as means ± SE of n = 6 to 12 replicates. ***: different from control, p < 0.0001. NS: no significant difference.

PLD being an upstream regulator of the mTOR pathway, we next assessed whether the activity of mTOR is required for the hypertrophic effect of PLD1 overexpression. To this end, we used the PP242 inhibitor, which blocks both mTORC1 and mTORC2 complexes [38]. In line with published work showing that mTORC1 is inhibited in muscle atrophy, we observed a marked reduction of myotube size and CK activity in myotubes treated by PP242 alone (Figure 8A,B). Moreover, we found the PP242 treatment to totally abolish the hypertrophic effects induced in myotubes by PLD1 overexpression, supporting the view that PLD1 acted through mTOR stimulation (Figure 8A,B).

**Figure 9:**
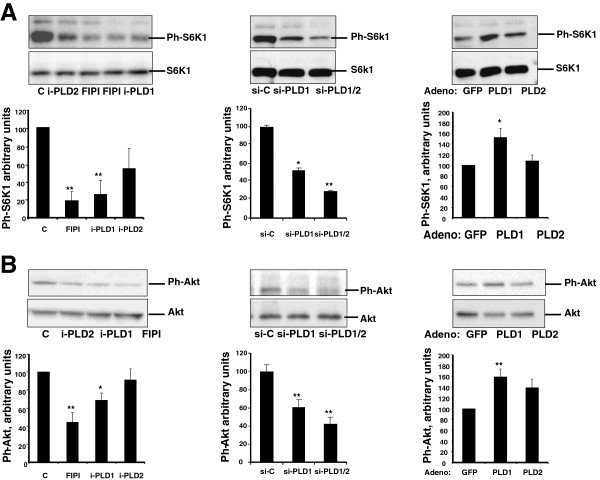
**PLD regulates mTOR signaling in myotubes. (A)** Left panel: myotubes were left untreated (C), or were treated with 0.5 μM FIPI, or with 100 nM of a PLD1-specific inhibitor (i-PLD1), or with 100 nM of a PLD2-specific inhibitor (i-PLD2). Middle panel: myotubes were transfected with control siRNA (siC), or with PLD1-siRNA (siPLD1), or with a siRNA directed against both PLD1 and PLD2 (siPLD1/2). Right panel: myotubes were infected with a GFP-adenovirus as a control, or with PLD1- or PLD2-adenovirus. Protein extracts were used to evaluate Phospho-Thr389/Thr412-S6K1 by immunoblotting. Quantification of the bands normalized to the total S6K1 amount is given in the diagrams, as means ± SE of n = 3 to 5 experiments. **(B)** Myotubes were treated as described above, and Ph-Ser473 Akt was detected by immunoblotting in the protein extracts. Quantification of the bands normalized to the total Akt amount is given in the diagrams as means ± SE of n = 3 to 7 experiments. **: different from control, p < 0.01; *: p < 0.05.

We further explored the influence of PLD on mTOR signaling by evaluating the consequences of PLD modulation on the phosphorylation of S6K1 and Akt, which are downstream effectors of, respectively, mTORC1 and mTORC2. Whereas PLD1 overexpression increased S6K1 phosphorylation, siRNA-mediated PLD depletion had the opposite effect. In the same line of observations, we found the PLD inhibitors able to decrease S6K1 phosphorylation, FIPI and the PLD1-specific inhibitor being more efficient than the PLD2-specific inhibitor (Figure 9A). Moreover, siRNA-mediated PLD1 depletion or PLD1 inhibition decreased Akt phosphorylation levels, whereas PLD1 overexpression had the opposite effect (Figure 9B). It is worth mentionning that PLD2 overexpression induced moderate, non significant, effects on S6K1 or Akt activation (Figure 9A,B). Together, these results suggest that, in L6 myotubes, PLD is involved in both mTORC1 and mTORC2 activation, mainly through its PLD1 isoform. We also observed that treating myotubes by dexamethasone or 1-butanol induced an inhibition of both S6K1 and Akt phosphorylation, therefore confirming that in atrophy-promoting conditions mTOR signaling is inhibited (Additional file 2). Furthermore, we verified that siRNA-mediated depletion of Rictor (and thus disruption of the mTORC2 complex) decreased the phosphorylation of Akt, confirming that Akt is a substrate for mTORC2 in L6 myotubes (Additional file 2).

## Discussion

Skeletal muscle displays a striking plasticity, mature muscle cells undergoing drastic changes in their size and specific protein content to adapt the tissue to different levels of mechanical stimulation or nutrient income, or to hypercatabolic pathological situations [39]. mTOR signaling is known to play a central role in the mechanisms that control muscle plasticity [15]. The involvement of PLD in muscle hypertrophy induced by mechanical loading has been hypothesized, due to the functional connection that exists between PLD activity and mTOR signaling. Mechanical stimuli have been shown to induce a PLD-dependent mTORC1 activation in isolated muscles, however the participation of PLD in the hypertrophic response was not demonstrated [24,25].

Here we report that, in differentiated myotubes, the suppression of PLD activity obtained by either addition of a primary alcohol or specific inhibitors, or by RNA interference, results in an atrophic effect, as evidenced by a size reduction and a decrease in the content in muscle proteins such as creatine kinase or MHC. Conversely, we observed that the overexpression of PLD is able to induce marked hypertrophic effects, showing that muscle cell size is positively regulated by PLD. In both the cases of PLD inhibition and overexpression, we observed that trophic effects depend on PLD1, rather than PLD2. Although these two PLD isoforms display a strong sequence homology, and are both dependent on PIP2 for their activity, they exhibit quite different regulatory properties and subcellular localizations. Whereas PLD1 has a low basal activity *in vitro* and is activated by small G proteins (ARF, Rho and Rac) and protein kinase C, PLD2 has a high basal activity and does not respond to the PLD1 activators. Moreover, under steady-state conditions, PLD1 has a predominently perinuclear location, whereas PLD2 is found at the plasma membrane, which suggests that the isoforms have different biological functions [1-3]. The respective participation of PLD1 and PLD2 in mTORC1 activation is still debated [7,8]. Thus, PLD1 was shown to be indispensable for amino-acid activation of mTORC1 [13]. Rheb, which is implicated in the activation of mTORC1, directly activates PLD1 [12]. However, PLD1 and PLD2 dominant negative mutants have both been found to suppress mTORC1 and mTORC2 activity [10], and PLD2 overexpression can activate mTORC1 [40]. Furthermore, PLD2 was reported to form with mTOR and Raptor a functional complex that is essential for mitogen stimulation of S6K1 [41]. Thus it appears that both PLD isoforms can be involved in mTOR regulation, depending on the cellular context. Although in our exerimental setting PLD2 inhibition tended to decrease S6K1 phosphorylation, and thus mTORC1 activity, this did not significantly affect myotube size, suggesting that the impact of PLD2 activity on mTOR is insufficient to regulate downstream pathways.

We also observed that PLD1 overexpression induces a hypertrophy of myofibres *in vivo*, similar to what observed in L6 myotubes. The ability of PLD1 overexpression to up-regulate cell size had been reported in non-muscle HEK293 cells [13]. Our results further establish that PLD1 is able to induce hypertrophy of differentiated muscle cells, and suggest that it may play a role in physiological situations that impact muscle mass. In this regard, PLD has been proposed to be a link between mechanical stimulation of muscle and mTORC1 activation resulting in hypertrophic response [42]. This hypothesis is supported by the co-localization that exists in muscle tissue between both PLD1 and PLD2 and the z-band protein α-actinin, z-band being considered a focal point for mechanical force transmission [24].

Our finding that PLD1 overexpression prevents the severe myotube atrophy induced by dexamethasone treatment shows that PLD1 has also a protective effect. This observation is further confirmed by the effects of PA, the product of PLD which directly binds to mTOR. Exogenous PA was indeed able to protect myotubes against atrophy induced by both dexamethasone and TNFα, indicating that the catalytic activity of PLD is required for its anti-atrophic effects. This was confirmed by our observation that the inhibition of PLD activity by FIPI suppresses both the hypertrophic and anti-atrophic effects of PLD1. Surprisingly, we did not observe a hypertrophic effect of exogenous PA when added alone to the myotubes (Figure 6). Therefore, it is likely that the subcellular site of PA accumulation is critical for its trophic effects, and that, in cells submitted to PLD1 overexpression PA accumulation occurs in a compartment that is inefficiently reached by exogenous PA. PA target(s) might become more sensitive to PA supply under atrophic conditions, and could be affected by lower concentrations of the compound, explaining why exogenous PA addition had an anti-atrophic effect.

The positive trophic effects of PLD1 or PA in basal conditions and in the presence of dexamethasone were both associated with a reduced expression of genes involved in muscle protein breakdown, Murf1, Atrogin-1 and Foxo3.

We addressed the mechanism by which PLD exerts its trophic effects by using PP242, a mTOR inhibitor directed at the catalytic site of the kinase, and that thus inhibits the activity of both mTORC1 and mTORC2 complexes. Interestingly, compared with rapamycin PP242 has been shown to more completely inhibit the phosphorylation of mTORC1 substrates (e.g. 4E-BP1) and mTORC2 substrates (e.g. Akt) [38]. PP242 treatment blocked PLD1 hypertrophic effects, showing that they rely on the activation of either mTORC1, or mTORC2, or of both complexes. This latter assumption is supported by the enhanced phosphorylation of both S6K1 and Akt observed in myotubes overexpressing PLD1, and by the decreased phosphorylation of these two effectors under PLD1 down-regulation or inhibition.

**Figure 10:**
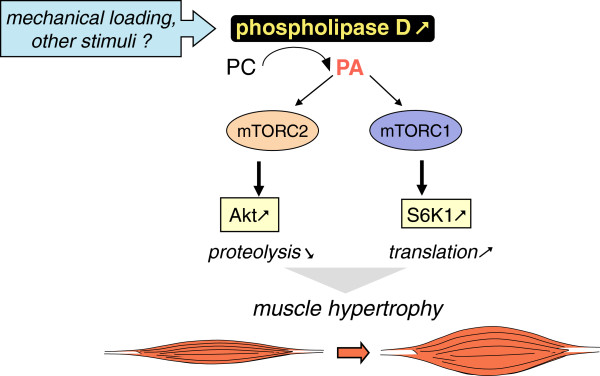
Schematic model for phospholipase D action on muscle tissue.

Protein homeostasis is under the control of the intricate network of the Akt/mTOR signaling pathway. Akt is a major inhibitor of proteolysis through the control of Foxo transcription factors. In muscle, Foxo factors regulate both the proteasome-dependent degradation of specific muscle proteins, and the autophagic proteolysis [37]. The mTORC2 complex formed by mTOR associated with Rictor is able to phosphorylate and activate Akt, whereas the mTORC1 complex formed by mTOR and Raptor is indirectly activated by Akt, through the phosphorylation of the tuberous sclerosis complex [6]. Activated mTORC1 is known to enhance protein translation through the phosphorylation of its substrates S6K1 and 4E-BP1, and to inhibit autophagy [6]. Thus, it is likely that the hypertrophic and anti-atrophic effects that PLD exerts on differentiated myotubes rely on the activation of both mTORC1 and mTORC2 complexes. This hypothesis is in agreement with the findings of Toschi et al. who showed that PLD and PA are required for the formation and activity of both mTORC1 and mTORC2 [10]. Studies carried out with transgenic mouse models have not discovered a role for mTORC2 in muscle mass regulation, since contrary to what observed in mice with mTORC1-deficient muscle, the animals with genetically disrupted mTORC2 in muscle do not display an obvious phenotype in standard conditions [19]. It is however conceivable that the muscles of these mTORC2 mutant animals develop altered trophic responses that would need to be explored upon exposure to chronic mechanical loading or atrophy-promoting treatments. Based on all these observations, we propose in Figure 10 a novel model depicting the action of phospholipase D within muscle tissue.

## Conclusions

Muscle atrophy occurs in a variety of pathological states such as cancer, renal insufficiency, diabetes and sepsis. The loss of skeletal muscle constitutes a major health problem as it leads to reduced mobility and quality of life, lowered response to treatments, and decreased life expectancy. Studies carried out on murine models of cancer cachexia have shown that reversing muscle loss dramatically prolongs animal survival, highlighting the usefulness of treatments preserving muscle mass [43]. The present work, by showing the protective effects of PLD and PA against dexamethasone- and TNFα-induced muscle cell atrophy points out the PLD pathway as a possible target for therapeutical interventions aiming at preserving muscle tissue in pathological situations. Importantly, the ability of stable phosphonate analogs of PA to activate mTORC1 signaling in cell cultures [44] suggests that these compounds could present a therapeutic potential which deserves further investigation.

## Methods

### Materials and reagents

ECL detection reagent was from Pierce Thermo Fisher Scientific (Brebières, France). Bradford protein assay was from Bio-Rad (Marnes-La-Coquette, France). Arginine-vasopressin (AVP), compound PP242, 5-Fluoro-2-indolyldeschlorohalopemide (FIPI), dioctanoyl-PA, dexamethasone and myosin heavy chain were purchased from Sigma-Aldrich (L’Isle-d’abeau, France). Selective inhibitors of PLD1 (CAY10593) and PLD2 (CAY10594) were supplied by Cayman Chemical Co. (Ann Arbor, USA). Recombinant rat TNFα was from Immunotools (Friesoythe, Germany). Anti-phospho-Thr389/Thr412-S6K1 antibody, anti-S6K1 antibody, anti-phospho-Ser473-Akt antibody and anti-Akt antibody (which recognize all three Akt isoforms) were from Cell Signaling Technology (Danvers, USA). Anti-sarcomeric myosin heavy chain MF-20 antibody was from Developmental Studies Hybridoma Bank, University of Iowa (Iowa City, USA). Anti-HA tag antibody was from Covence (Rueil-Malmaison, France). Anti-laminin antibody was from Sigma-Aldrich. HRP-conjugated anti-mouse- and anti-rabbit-IgG antibodies were from Jackson Immunoresearch Laboratories (Soham, UK).

### Cell culture

L6 myoblasts were maintained in Dulbecco’s modified Eagle’s medium (DMEM) with 4.5 g/l glucose, supplemented with 10% (v/v) fetal bovine serum at 37°C and 5% CO_2_. To induce differentiation, cells were seeded at a density of 5.10^5^ cells per well in 6-well plates, grown to confluence, shifted to DMEM supplemented with 1% fetal bovine serum and 10^-7^ M AVP, and cultured for 5 days. The obtained myotubes were then treated with the appropriate agent for 2 days, or with 15 ng/mL recombinant rat TNFα for 3 days to induce atrophy. Dioctanoyl-PA stock solution was obtained by solubilizing the compound in Tris pH 8 buffer at a concentration of 50 mM.

### Short interfering RNA (siRNA) transfection

The siRNA used were targeted to rat PLD1 sequence 5’-AAGTTAAGAGGAAATTCAAGC- 3’, rat PLD2 sequence 5’-GACACAAAGTCTTGATGAG-3’, rat PLD1/2 sequence 5′-GAAATGGAGCCATCCCTCA-3′. Control siRNA was purchased from Eurogentec (Angers, France). siRNAs targeting Rictor and Raptor have been described in [22].

The transfection of siRNAs was performed using Hiperfect reagent (Qiagen, Courtaboeuf, France) with 50 nM siRNA for 48 hours; the medium was changed after 24 hours of transfection.

### Adenoviral constructions and cell infection

Recombinant adenoviral constructs carrying the cDNA of interest (hHA-PLD1b, hHA-PLD2 or GFP) were generated as previously described [45]. Infections of myotubes were performed at a multiplicity of infection of 100 (with regard to initial myoblast number) in complete medium. After 24 hours of incubation in the presence of viral particles, the medium was changed and cells were cultured for additional 24 hours. Under these conditions, most of the cells were positive for GFP when infected with a GFP expressing adenovirus.

### Measurement of myotube area

Differentiated myotubes were fixed with 3.7% formaldehyde for 20 minutes at room temperature, permeabilized with 0.1% Triton for 10 minutes, and unspecific labeling was blocked with 1% BSA for 20 minutes. Anti-Myosin MF-20 antibody (1:50) was incubated for 1 hour. After washing by 1% BSA in PBS, rhodamine-conjugated anti-mouse IgG antibody was added diluted 1:500 in 1% BSA and incubated for 1 hour. Nuclei were stained with 1 μg/mL 4,5-diamidino-2-phenylindole (DAPI) for 3 minutes. Cells were examined by immunofluorescence microscopy with an Axiovert 200 microscope, and images acquired using Axiovision 4.1 software (Carl Zeiss, Göttingen, Germany). Differentiated myotubes, but not myoblasts, were evenly labeled on their entire surface. Their area was measured by the method of Sultan et al. [30], using NIH Image J software. To verify that the various treatments did not induce a cell loss leading to underestimation of myotube area, we evaluated the number of DAPI-stained nuclei in the entire fields, and found no significant loss of nuclei in atrophy-promoting conditions (not shown).

### Assay of creatine kinase activity

Cells were scraped with 500 μl of ice-cold lysis buffer containing 20 mM Tris–HCl, 100 mM NaCl, 1% Triton and protease inhibitor cocktail (pH 7.6). Lysates were kept on ice during 15 minutes and cleared by centrifugation at 13,000 g for 15 minutes. The creatine kinase activity assay was performed by using a CK – NAC LD B kit from Sobioda (Montbonnot, France), which allows to monitor at 340 nm the kinetics of NADPH formation. The assay was performed in 96-well plates, with 4 μL of sample and 100 μL of reagent per well, for 20 minutes at 30°C.

### ELISA of myosin heavy chain

Cells were scraped in 300 μL ice-cold RIPA buffer, vortexed and centrifuged at 10,000 g for 10 minutes. The assay was carried out in 96-well plates on 50 μL of 1:50 diluted samples. The wells were evaporated to dryness overnight at 37°C and washed twice with cold PBS, using an automatic plate washer (ELx50 Autostrip Washer from Bio-Tek Instruments, Inc.). Unspecific binding sites were saturated with 100 μL of 0.3% BSA in PBS for 30 minutes at 37°C. Samples were then incubated with 50 μL MF-20 antibody diluted 1:100 in PBS, for 1 hour at 37°C. After a new washing step in 0.2% Tween 20 in PBS, incubation with 50 μL of secondary HRP-conjugated anti-mouse IgG antibody diluted 1:3000 was performed for 1 hour at 37°C. Plates were washed 5 times, 50 μL of TMB substrate (Sigma-Aldrich) were added to each well, and 50 μL 0.5 N H_2_SO_4_ were added after 5 min to stop color reaction. Optical Density was read at 450 nm. A standard curve was obtained with purified myosin heavy chain.

### Western blotting

Cells were lyzed as for CK assay, in the presence of 10 mM sodium pyrophosphate, 10 mM glycerophosphate, 50 mM NaF, 1.5 mM Na_3_VO_4_. Cell lysates were analyzed by SDS/PAGE, and proteins were transferred onto PVDF membranes blocked with 5% BSA in Tris-buffered saline/0.1% Tween 20, and incubated with appropriate antibodies following manufacturers’ recommendations. Immunoblots were revealed with ECL detection system (Pierce) and quantified with Image J software. SDS-PAGE was performed using 10% polyacrylamide gels for S6K1 and Akt. In the case of PLDs, samples were subjected to SDS-PAGE on 8% polyacrylamide gels, in the presence of 4 M urea.

### *In vivo* experiments

5 week-old male BALB/c mice were obtained from Charles River France. Animals were housed in the animal facility under standard conditions. Adenovirus encoding PLD1 (10^9^ infectious units in 100 μL PBS) were injected in the right gastrocnemius, the left gastrocnemius being injected with the same amount of control GFP-encoding adenovirus. The animals were sacrificed 10 days post-injection, gastrocnemius muscles were dissected from both hind-limbs, frozen in liquid N_2_-cooled isopentane and stored at −80°C for either histological or molecular analyses. Muscle cryo-sections (10 μm) were stained with Hematoxylin-Eosin, and fibre cross sectional areas (at least 300 fibres per muscle) were measured by using NIH Image J software. Alternatively, sections from the PLD1-injected muscles were immuno-labeled for laminin and for HA-tag, to respectively determine fibre outline and detect PLD1-expressing fibres. Fibre CSA was determined as above.

Mice were treated in strict accordance to the guidelines of the Institutional Animal Care and Use Committee and to relevant national and European legislation, throughout the experiments.

### Reverse transcription and real-time PCR

Total RNA was isolated from L6 myotubes using Trizol Reagent (Life Technologies, Saint-Aubin, France). 1 μg of total RNA was used for reverse transcription, in the presence of 100 U Superscript II (Life Technologies), random hexamers and oligo dT. Real-time PCR was performed with Fast Start DNA Master Sybr green kit using Rotor-Gene 6000 (Corbett research, Mortlake, Australia). Data were analyzed with LightCycler software (Roche Diagnostics, Meylan, France) and normalized to TATA box binding protein (TBP) housekeeping gene transcripts. Specific sense and antisense primers used for amplification were as follows: rPLD1 sense: GGTCAGAAAGATAACCCAGG, rPLD1 anti-sense: GAAGCGAGACAGCGAAATGG; rPLD2 sense: TTGCTGGCTGTGTGTCTGGC, rPLD2 antisense: GGACCTCCAGAGACACAAAG; hPLD1 sense: AAAGCGTGACAGTGAAATGG, hPLD1 anti-sense: GGCCATCAAGATAGCCAAGG; Atrogin-1 sense: CTCTGCCAGTACCACTTCTC, Atrogin-1 anti-sense: ATGGTCAGTGCCCCTCCAGG; Murf1 sense: TGCATCTCCATGCTGGTGGC, Murf1 anti-sense: CTTCTTCTCGTCCAGGATGG; Foxo3a sense: GAGAGCAGATTTGGCAAAGG, Foxo3a anti-sense: CCTCATCTCCACACAGAACG; TBP sense: TGGTGTGCACAGGAGCCAAG, TBP anti-sense: TTCACATCACAGCTCCCCAC.

### Statistical analyses

The statistical significance of data was assessed by ANOVA and Fisher test, using StatView software.

## Abbreviations

PLD: Phospholipase D; PA: Phosphatidic acid; mTOR: Mammalian target of rapamycin; mTORC1: mTOR complex 1; mTORC2: mTOR complex 2; TNFα: Tumor necrosis factor α; MHC: Myosin heavy chain; FIPI: 5-Fluoro-2-indolyl-deschlorohalopemide; CK: Creatine kinase.

## Competing interests

The authors declare that they have no competing interests.

## Authors’ contributions

RJ and JDL performed most of the experiments, and analyzed the data. SC, VE, and CD participated in experiments. FN, PB, HV and EL participated in the coordination of the study and critically revised the manuscript. GN designed the research, analyzed the data, and wrote the article. All the authors read and approved the final manuscript.

## Supplementary Material

Additional file 1 **Comparison of the CSA of PLD1-expressing and non expressing fibres in PLD1 adenovirus-injected muscles.** (A) Muscle transversal sections were immuno-labeled for laminin and for HA-tagged PLD1. (B) The distributions of CSA of PLD1-expressing and non expressing fibres are shown. Click here for file

Additional file 2 **Effects of various agents on the phosphorylation of mTORC1 substrate S6K1 and mTORC2 substrate Akt.** (A) Myotubes were treated for 2 days with 0.1% 1-butanol or 0.1% t-Butanol as a control. (B) Myotubes were left untreated, or treated for 2 days with 50 μM or 100 μM dexamethasone. (C) Myotubes were transfected for 2 days with control siRNA (si-C), or siRNA directed against Raptor (si-Rapt), or Rictor (si-Rict). Phospho-Thr389/412-S6K1, total S6K1, Ph-Ser473-Akt, total Akt, were then detected by immunoblotting. Click here for file
