# Supplementary material for: Phospholipase D regulates the size of skeletal muscle cells through the activation of mTOR signaling
Source: Cell Commun Signal. 2013 Aug 2;11:55. doi: 10.1186/1478-811X-11-55 (PMC3765503; doi:10.1186/1478-811X-11-55)
Supplement: Additional file 1 — Comparison of the CSA of PLD1-expressing and non expressing fibres in PLD1 adenovirus-injected muscles. (A) Muscle transversal sections were immuno-labeled for laminin and for HA-tagged PLD1. (B) The distributions of CSA of PLD1-expressing and non expressing fibres are shown. [file 1478-811X-11-55-S1.pdf]

Phospholipase D regulates the size of skeletal muscle cells through the activation of mTOR signaling

Rami Jaafar [1], Joffrey De Larichaudy [1], Stéphanie Chanon [1], Vanessa Euthine [1], Christine Durand [1], Fabio Naro [2], Philippe Bertolino [3] , Hubert Vidal [1], Etienne Lefai [1], Georges Némoz [1]

[1] Lyon 1 University, INSERM U1060, CarMeN Laboratory, Institut National de la Recherche Agronomique USC1235, F-69600 Oullins, France. [2] Istituto Interuniversitario di Miologia and Dipartimento di Istologia ed Embriologia Medica, Università di Roma-La Sapienza, 00161 Roma, Italy. [3] Centre de Recherche en Cancérologie de Lyon, INSERM U1052, CNRS UMR 5286, 69008 Lyon, France.

A

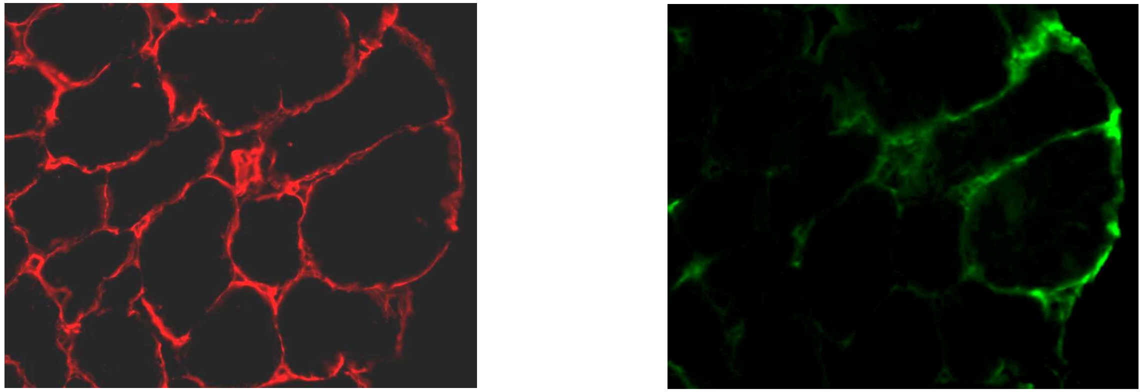

B

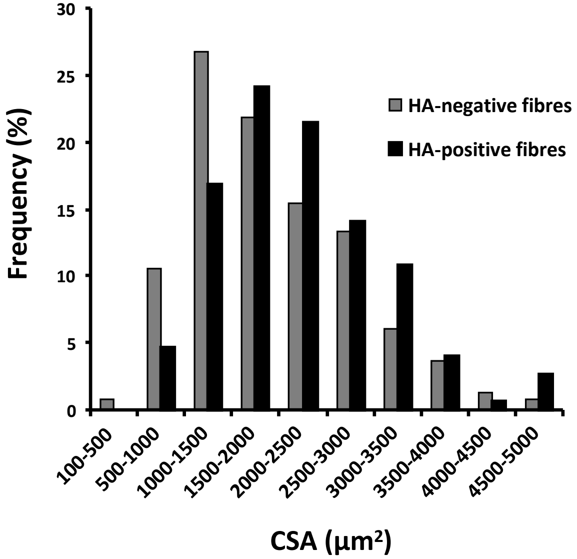

Additional file 1. Comparison of the CSA of PLD1-expressing and non expressing fibres in PLD1 adenovirus-injected muscles. (A) Muscle transversal sections were immuno-labeled for laminin and for HA-tagged PLD1. (B) The distributions of CSA of PLD1 -expressing and non expressing fibres are shown.
