# Supplementary material for: Phospholipase D regulates the size of skeletal muscle cells through the activation of mTOR signaling
Source: Cell Commun Signal. 2013 Aug 2;11:55. doi: 10.1186/1478-811X-11-55 (PMC3765503; doi:10.1186/1478-811X-11-55)
Supplement: Additional file 2 — Effects of various agents on the phosphorylation of mTORC1 substrate S6K1 and mTORC2 substrate Akt. (A) Myotubes were treated for 2 days with 0.1% 1-butanol or 0.1% t-Butanol as a control. (B) Myotubes were left untreated, or treated for 2 days with 50 μM or 100 μM dexamethasone. (C) Myotubes were transfected for 2 days with control siRNA (si-C), or siRNA directed against Raptor (si-Rapt), or Rictor (si-Rict). Phospho-Thr389/412-S6K1, total S6K1, Ph-Ser473-Akt, total Akt, were then detected by immunoblotting. [file 1478-811X-11-55-S2.pdf]

Phospholipase D regulates the size of skeletal muscle cells through the activation of mTOR signaling

Rami Jaafar [1], Joffrey De Larichaudy [1], Stéphanie Chanon [1], Vanessa Euthine [1], Christine Durand [1], Fabio Naro [2], Philippe Bertolino [3], Hubert Vidal [1], Etienne Lefai [1], Georges Némoz [1]
[1] Lyon 1 University, INSERM U1060, CarMeN Laboratory, Institut National de la Recherche Agronomique USC1235, F-69600 Oullins, France. [2] Istituto Interuniversitario di Miologia and Dipartimento di Istologia ed Embriologia Medica, Università di Roma-La Sapienza, 00161 Roma, Italy. [3] Centre de Recherche en Cancérologie de Lyon, INSERM U1052, CNRS UMR 5286, 69008 Lyon, France.

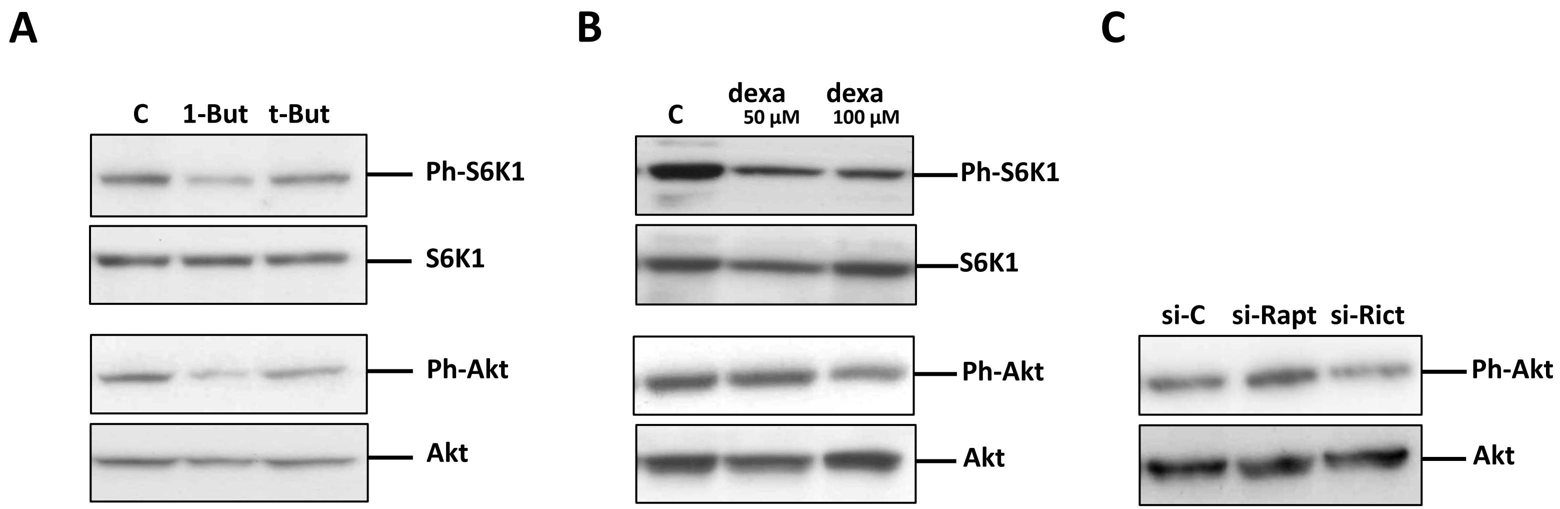

Additional file 2. Effects of various agents on the phosphorylation of mTORC1 substrate S6K1 and mTORC2 substrate Akt. (A) Myotubes were treated for 2 days with 0.1% 1-butanol or 0.1% t-Butanol as a control. (B) Myotubes were left untreated, or treated for 2 days with 50 µM or 100 µM dexamethasone.(C) Myotubes were transfected for 2 days with control siRNA (si-C), or siRNA directed against Raptor (si-Rapt), or Rictor (si-Rict). Phospho-Thr389/412-S6K1, total S6K1, Ph-Ser473-Akt, total Akt, were then detected by immunoblotting.
